# 626. Carbapenem-resistant Enterobacterales surveillance and increase in New Delhi Metallo-β-lactamase producing CRE–New York City, 2019–2024

**DOI:** 10.1093/ofid/ofaf695.193

**Published:** 2026-01-11

**Authors:** Katelynn Devinney, William Greendyke, Karen Alroy, Nicole Burton, Addie Crawley, Cherry-Ann Da Costa-Carter, Molly M Kratz, Ying Lin, Tristan D McPherson, Jorge Montfort Gardeazabal, Thomas Portier, Celina Santiago, Ulrike Siemetzki-Kapoor, Matthew Sullivan, Rain Wiegartner

**Affiliations:** NYC Department of Health and Mental Hygiene, Queens, New York; NYC Department of Health and Mental Hygiene, Queens, New York; NYC Department of Health and Mental Hygiene, Queens, New York; NYC Department of Health and Mental Hygiene, Queens, New York; NYC Department of Health and Mental Hygiene, Queens, New York; NYC Department of Health and Mental Hygiene, Queens, New York; NYC Department of Health and Mental Hygiene, Queens, New York; NYC Department of Health and Mental Hygiene, Queens, New York; New York City Department of Health and Mental Hygiene, New York, NY; NYC Department of Health and Mental Hygiene, Queens, New York; NYC Department of Health and Mental Hygiene, Queens, New York; NYC Department of Health and Mental Hygiene, Queens, New York; NYC Department of Health and Mental Hygiene, Queens, New York; NYC Department of Health and Mental Hygiene, Queens, New York; NYC Department of Health and Mental Hygiene, Queens, New York

## Abstract

**Background:**

Carbapenem-resistant Enterobacterales (CRE) infections are an urgent threat; *Klebsiella pneumoniae* carbapenemase (KPC) has been the predominant carbapenemase (CP) in the USA since 1996. New-Delhi metallo-β-lactamase (NDM) is less common in the USA and confers resistance to antibiotics used to treat KPC+CRE. The New York City (NYC) Health Code was amended in 2018 to mandate laboratory reporting of CRE organisms with susceptibility and CP results. We aimed to describe CRE cases during 2019–2024, including CP frequency.

**Figure:**
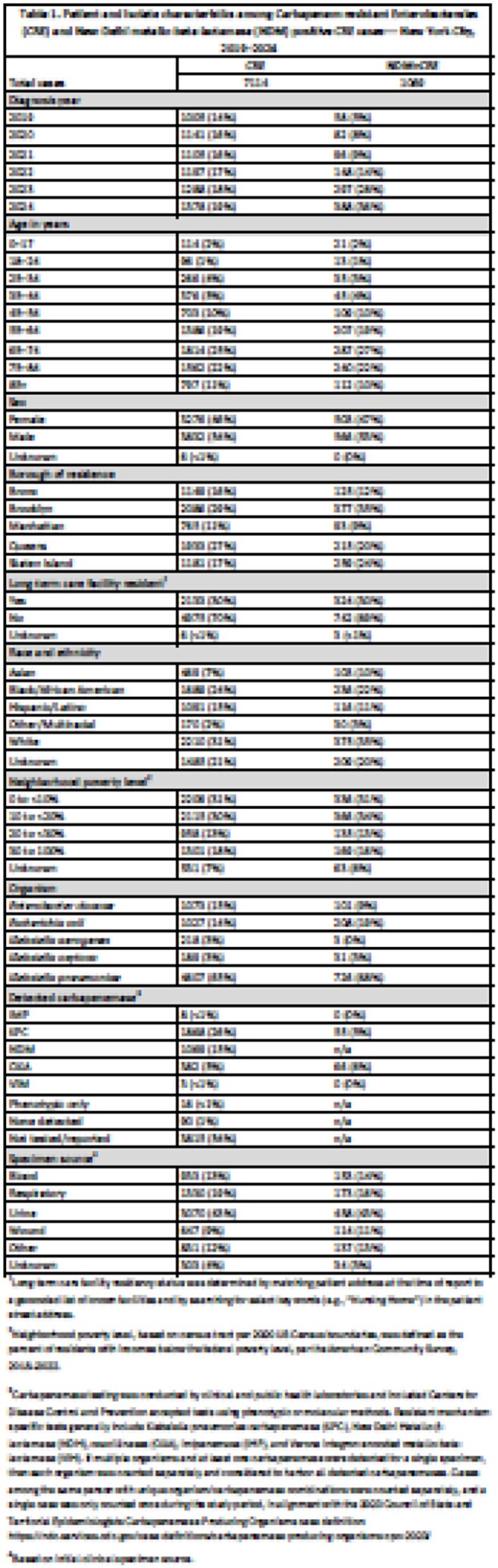

**Figure:**
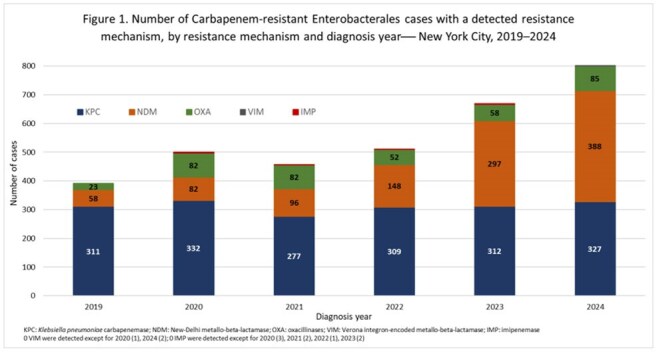

**Methods:**

CRE cases were defined as clinical specimens collected among NYC residents during 2019–2024 that were positive for *Escherichia coli*, *K. pneumoniae*, *K. aerogenes*, *K. oxytoca*, or *Enterobacter cloacae,* and carbapenem resistant or harbored a CP. We assessed patient and isolate characteristics of CRE cases and calculated annual incidence rates.

**Figure:**
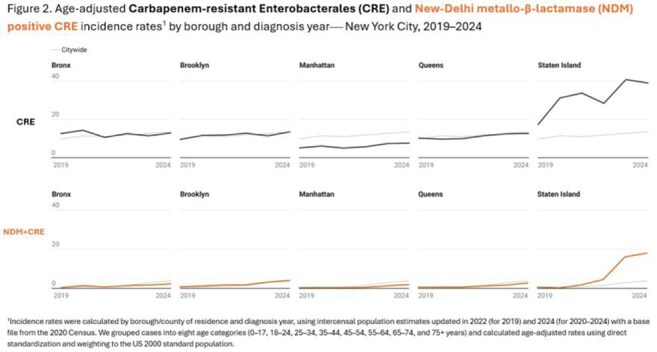

**Results:**

Among 7,114 reported patients with CRE, the median age was 68 years, and 54% were male. CRE cases increased from 1,005 in 2019 to 1,378 in 2024 (Table 1). The percentage of CRE cases with CP results ranged from 40% to 54%. NDM surpassed KPC as the most frequently reported CP in 2024 (Figure 1). Overall, 30% of patients with CRE and NDM+CRE were long-term care facility (LTCF) residents, peaking for NDM+CRE in 2021 (38%) and decreasing to 25% in 2024. The most frequent organism was *K. pneumoniae* for CRE (65%) and NDM+CRE (68%); urine was the most common specimen source (43% for both). Age-adjusted incidence rates increased in the borough of Staten Island for CRE (from 17 to 39 cases/100,000 residents) and NDM+CRE (from < 1 to 18 cases/100,000 residents) (Figure 2).

**Conclusion:**

The increase in CRE incidence since 2019 may be in part due to improved reporting; however, increasing NDM+CRE incidence is unlikely to be solely driven by increased testing or reporting, given the relative stability in other CP. The proportional decrease in NDM+CRE cases in LTCF residents suggests that increased NDM+CRE incidence possibly originated with healthcare clusters and outbreaks that led to sustained transmission beyond hospital and LTCF settings. We are implementing whole genome sequencing of CRE isolates to better understand transmission in NYC. Providers should consider rising NDM incidence when treating CRE.

**Disclosures:**

All Authors: No reported disclosures

